# Research Advances in Electrospun Nanofiber Membranes for Non-Invasive Medical Applications

**DOI:** 10.3390/mi15101226

**Published:** 2024-09-30

**Authors:** Junhua Wang, Chongyang You, Yanwei Xu, Tancheng Xie, Yi Wang

**Affiliations:** 1 College of Mechanical and Electrical Engineering, Henan University of Science and Technology, Luoyang 471003, China; wangjh@haust.edu.cn (J.W.); youchongyang9999@163.com (C.Y.); xuyanweiluoyang@163.com (Y.X.); 2 Department of Mechanical Engineering, Beijing University of Technology, Beijing 100124, China; 3 Henan Intelligent Manufacturing Equipment Engineering Technology Research Center, Luoyang 471003, China; 4 Henan Engineering Laboratory of Intelligent Numerical Control Equipment, Luoyang 471003, China

**Keywords:** non-invasive, electrospinning, nanofibers, health

## Abstract

Non-invasive medical nanofiber technology, characterized by its high specific surface area, biocompatibility, and porosity, holds significant potential in various medical domains, including tissue repair and biosensing. It is increasingly becoming central to healthcare by offering safer and more efficient treatment options for contemporary medicine. Numerous studies have explored non-invasive medical nanofibers in recent years, yet a comprehensive overview of the field remains lacking. In this paper, we provide a comprehensive summary of the applications of electrospun nanofibers in non-invasive medical fields, considering multiple aspects and perspectives. Initially, we introduce electrospinning nanofibers. Subsequently, we detail their applications in non-invasive health, including health monitoring, personal protection, thermal regulation, and wound care, highlighting their critical role in improving human health. Lastly, this paper discusses the current challenges associated with electrospun nanofibers and offers insights into potential future development trajectories.

## 1. Introduction

As science and technology rapidly advance and society increasingly demands a higher quality of life and health, research into non-invasive medical technologies and materials is becoming increasingly central to the medical and healthcare sectors. Non-invasive medical technologies eliminate the need for traditional surgical procedures, reducing infection risks, accelerating recovery, and enabling more gentle and less burdensome treatments for a variety of diseases. This greatly improves patients’ quality of life and their treatment experience [1]. The choice and efficacy of non-invasive medical materials are crucial. Traditional materials often do not meet the precision demands of modern medicine, biocompatibility, and multifunctionality. The advent of nanofiber materials presents a promising solution in this context [2]. Nanofibers, defined by their minute size, high surface-area-to-volume ratio, and distinctive physicochemical properties, show significant potential in applications such as drug delivery, tissue engineering, and biosensing. The main production techniques for nanofibers include stretching, deposition, self-assembly, and phase separation, yet they are often impeded by complexity, inefficiency, and stringent environmental needs, limiting their wider adoption. As a result, electrospinning has become an essential innovation within this domain [3].

Electrospinning, an efficient nanofiber fabrication process, involves stretching a polymer solution or melt into nanofibers by using a high-voltage electric field [4], representing an interdisciplinary convergence of physics, chemistry, and fluid dynamics. This contemporary nanotechnology offers rapid, controllable, cost-effective, and scalable production, showcasing considerable benefits in fabricating micro/nanofibers with consistent or tailored microstructures [5]. It can produce nanofibers with a high surface-area-to-volume ratio and fine diameter, which improve both drug loading and release efficiency as well as drug efficacy. Benefits of the technology are further highlighted by the versatility of its raw materials, encompassing natural polymers, synthetic polymers and inorganic substances. By fine-tuning formulations and process variables, nanofibers can be tailored for specific applications. The simplicity of electrospinning operations and their suitability for continuous production contribute to lowering costs and boosting efficiency, addressing the extensive medical need for high-performance nanomaterials.

**Figure 1:**
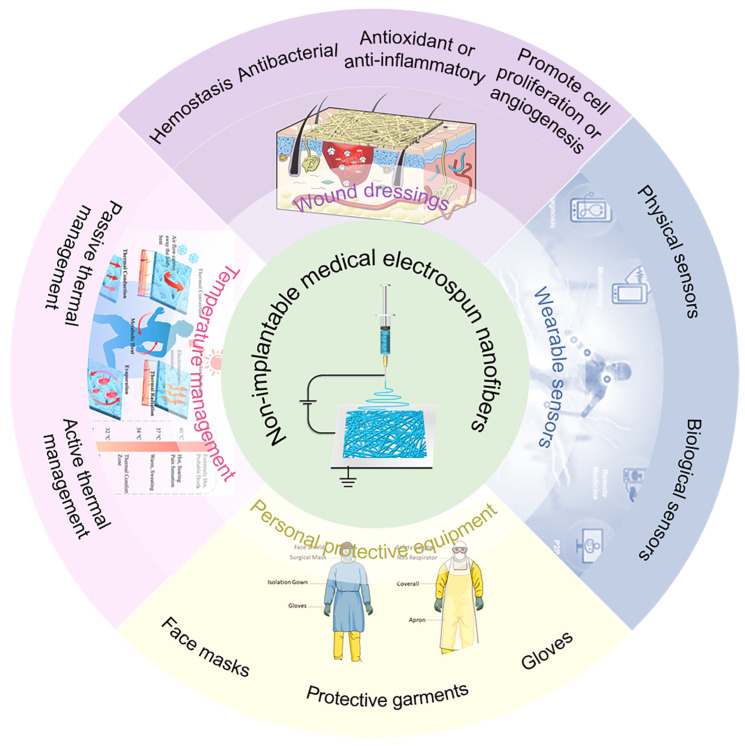
Application of electrospun nanofibers in non-invasive medical applications. Reproduced with permission [11]. Copyright 2022 John Wiley and Sons Publications. Reproduced with permission [12]. Copyright 2023 Springer Nature Publications. Reproduced with permission [13]. Copyright 2023 American Chemical Society Publications.

Numerous reviews on electrospun nanofibers have been published [6,7,8,9,10], yet few have offered a comprehensive overview of their applications in the non-invasive medical sector. This article systematically reviews and analyzes domestic and international research to offer a detailed summary of electrospun nanofibers’ advancements in non-invasive medical treatments, encompassing human health diagnostics, personal protective gear, thermal regulation, and wound care, as illustrated in Figure 1. It highlights the significant role of electrospun nanofibers in enhancing human health. Additionally, this article delves into the challenges and prospective directions within this field. This analytical review aims to offer insights and inspiration to researchers, fostering further exploration and broader adoption of electrospinning nanofiber technology in non-invasive medicine and healthcare.

## 2. Electrospun Nanofibers

Electrospinning is an efficient, economical method for preparing nanofibers and membranes with diverse structures. A key advantage of electrospinning is its ability to produce nanofibers featuring a high specific surface area, tunable pore structures, excellent mechanical properties, and good biocompatibility [14]. Furthermore, electrospun nanofibers can be modified or doped with nanoparticles to confer specific functionalities like antimicrobial activity or electrical conductivity. They hold promise for applications in drug delivery [15], catalysis [16], sensing [17], filtration [18], tissue engineering [19], wearable electronics [20], and flexible sensing [21].

### 2.1. Introduction to Electrospinning

**Figure 2:**
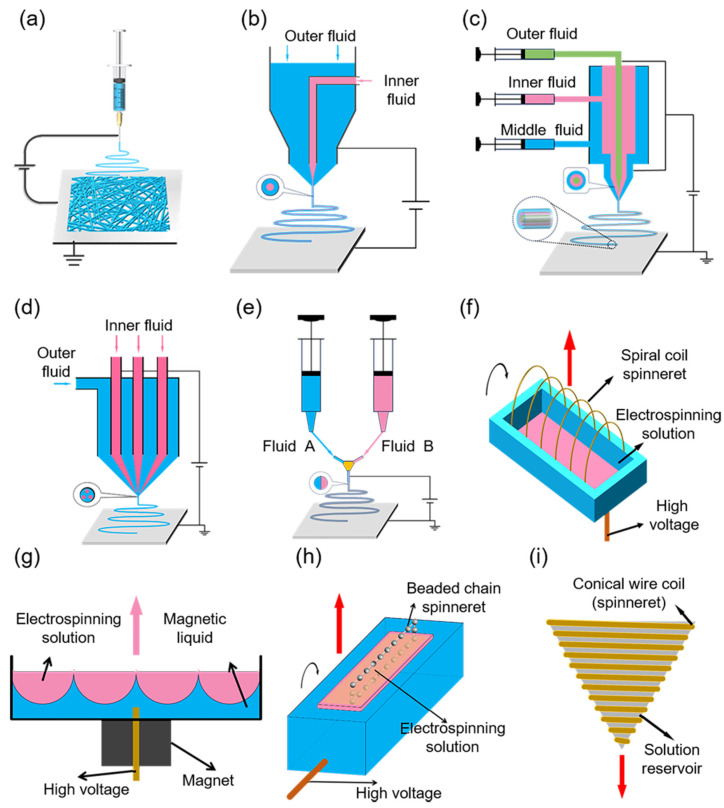
Schematic diagram of electrospinning. Needle-based electrospinning: (**a**) uniaxial electrospinning; (**b**) coaxial electrospinning; (**c**) triaxial electrospinning; (**d**) multifluidic electrospinning; (**e**) side-by-side electrospinning. Reproduced with permission [49]. Copyright 2019 Elsevier Publications. (**f**–**i**) Needle-free electrospinning generators. Reproduced with permission [5]. Copyright 2023 Elsevier Publications.

Electrospinning devices mainly consist of a high-voltage DC power supply, a spinneret, an injection pump, and a ground collector [5], as illustrated in Figure 2a. Based on the spinneret design, electrospinning is divided into two categories: needle-based and needleless. Each method has its pros and cons; needle-based electrospinning produces fine and uniform fibers, but the needles can easily clog and are difficult to clean; needle-free electrospinning enhances nanofiber yield, though the fibers are thicker and exhibit less uniformity.

#### 2.1.1. Needle-Based Electrospinning

Needle-based electrospinning is divided into single-needle and multi-needle methods, with the single-needle category encompassing single-axis, coaxial, triaxial, multi-fluid, and side-by-side electrospinning.

In 1902, Cooley and Morton introduced the first and most prevalent method of electrospinning, uniaxial electrospinning, derived from electrospraying [22]. Uniaxial electrospinning offers simplicity and low cost, enabling the production of fibers from micrometers to nanometers in diameter, with applications in biomedical materials [23], drug delivery [24], and materials science [25]. However, it faces limitations, including a limited range of fiber structures and functions, challenges in constructing complex 3D cellular architectures [26], the need for enhanced production efficiency, and further research into fiber morphology control [27].

In 2003, Sun et al. [28] developed coaxial electrospinning (Figure 2b), an advancement of the traditional electrospinning method. Coaxial electrospinning effectively constructs core-shell structured nanofibers in a fast and controllable manner [29]. It offers the ability to create nanofibers with complex structures and functions, including hollow types [30], and enables the combination of materials with varying properties. However, the complexity of the technique and its stringent parameter requirements can be challenging [31]. Coaxial electrospinning has been extensively applied in medical drug delivery systems [32], tissue engineering scaffolds [33], and biosensors [34], significantly advancing medical technology.

Triaxial electrospinning (Figure 2c), an advanced form of electrospinning, enables the concurrent spinning of multiple solutions with distinct properties, using a needle equipped with three concentric capillaries [35]. Not only is this method capable of producing nanofibers with more complex structures and richer functions (such as fibers with multilayered shell-and-core structures or special morphologies), but it also makes full use of the properties of different polymers to achieve complementary and optimized performance. This technology combines the advantages of multiple polymers and enhances the fiber’s performance, with potential applications in the fields of drug release, tissue engineering, and high-performance composites [35,36]. However, with the increase in the number of spools, the complexity and operational difficulty of the equipment increases, which puts higher demands on the precise control of spinning parameters.

Multifluid electrospinning (Figure 2d) represents an advanced nanofiber fabrication technique. Simultaneously introducing multiple fluids allows the creation of nanofibers with intricate structures and multiple functionalities [37]. This technology facilitates precise control over the ratio and distribution of nanofiber components and enables the construction of special structures like core–shell, hollow, and Janus, conferring unique physical, chemical, and biological properties to the fibers. However, controlling numerous fluid parameters raises the technical complexity and production costs, and fiber properties can vary due to multiple factors [38].

Side-by-side electrospinning (Figure 2e) is an advanced electrospinning technique employing parallel spinnerets or nozzles to concurrently spin multiple distinct solutions, yielding nanofibers with dual- or multi-component structures [39,40]. This technique enhances the flexibility and versatility of nanofiber production, enabling the fabrication of complex nanomaterials with diverse structures and functions in one step, with promising applications in sensor technology, medical materials, and other areas [41].

Multi-needle electrospinning is globally recognized as the simplest large-scale nanofiber production technology [42]. Compared with traditional single-needle electrospinning, multi-needle systems not only offer higher productivity but also allow for the use of various polymer solutions [43], enabling the fabrication of multi-component or blended nanofibrous membranes. However, narrow spacing between needles can lead to charge repulsion in the ejected jet from the Taylor cone, complicating the process. This can cause instability in the needle droplets, leading to clogging or dripping and impacting the uniformity of fiber diameters.

#### 2.1.2. Needleless Electrospinning

To enhance electrospinning efficiency, multiple needles can be employed [44], although they may experience electrostatic interference within the high-voltage electric field. The complexity of needle systems can also lead to clogging, limiting the industrial applicability of needle-based electrospinning. To address these issues, researchers have developed needleless spinning devices, focusing on creating numerous jet excitation points on the surface of the polymer solution. Recently, researchers have introduced various needleless electrospinning techniques, such as plate-edge, bowl, bead-chain, and conical filament methods [45,46,47,48], as illustrated in Figure 2f–i. Needle-free electrospinning significantly enhances production efficiency compared with its needle-based counterpart. However, the stability and controllability of this technology are considerably lower than needle electrospinning. Additionally, as open-type equipment, it is more environmentally sensitive. The volatility of the polymer solution, air humidity levels, and the variability of stretching positions make it challenging to achieve a uniform industrial electrospun film morphology. Thus, laboratory-based research continues to favor needle-type equipment [5].

#### 2.1.3. Available Biomaterials

To date, many polymers have been used in electrospinning. These can be roughly categorized into natural polymers, synthetic polymers, multi-component composite polymers, and inorganic/organic composite materials [50]. Commonly used natural polymers include collagen, gelatin, silk fibroin, spider silk protein, chitosan, and alginate; synthetic polymers mainly include poly(ε-caprolactone) (PCL), poly(lactic acid) (PLA),poly(lactic-co-glycolic acid) (PLGA), poly(ethylene glycol)-co-poly(d, l-lactide) (PELA), poly(ethylene glycol)-ploy(ε-caprolactone) (PEG-PCL), polyurethane (PU), polyvinyl alcohol (PVA), polyvinyl pyrrolidone (PVP), and polyethylene oxide (PEO), among others [6,8].

### 2.2. Structural Diversity of Nanofibers

#### 2.2.1. Structural Diversity of Single Nanofibers

Homogeneous nanofibers: Homogeneous nanofibers, characterized by a smooth surface and uniform cross-section [51], are the most fundamental type of nanofibers produced by electrospinning. For instance, Hajieghrary et al. [52] utilized cellulose nanofibers in varying concentrations to enhance the electrospinning properties of gelatin solutions. Scanning electron microscopy revealed that all composite nanofibers exhibited a uniformly ordered, continuous structure, free from breakage or bead defects.

**Figure 3:**
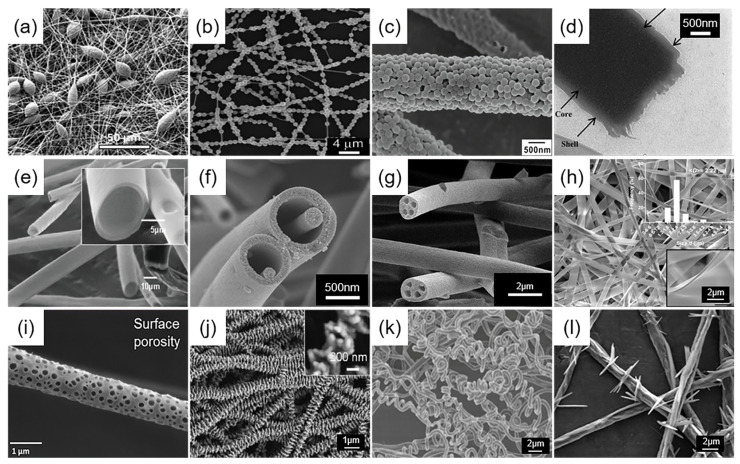
Diversity of single nanofibers. (**a**) Beaded fibers. Reproduced with permission [53]. Copyright 2023 Elsevier Publications. (**b**) Necklace-like fibers. Reproduced with permission [54]. Copyright 2010 American Chemical Society Publications. (**c**) Colloidal fibers. Reproduced with permission [55]. Copyright 2012 American Chemical Society Publications. (**d**) Core–shell fibers. Reproduced with permission [73]. Copyright 2016 Springer Nature Publications. (**e**) Hollow fibers. Reproduced with permission [58]. Copyright 2008 John Wiley and Sons Publications. (**f**) Nanowire-in-microtube structured fibers. Reproduced with permission [59]. Copyright 2010 American Chemical Society Publications. (**g**) Multichannel fibers. Reproduced with permission [74]. Copyright 2022 Springer Nature Publications. (**h**) Ribbon fibers. Reproduced with permission [62]. Copyright 2017 Elsevier Publications. (**i**) Porous fibers. Reproduced with permission [65]. Copyright 2018 Elsevier Publications. (**j**) Nanostructure fibers mimicking bone. Reproduced with permission [68]. Copyright 2013 American Chemical Society Publications. (**k**) Spiral fibers. Reproduced with permission [69]. Copyright 2019 John Wiley and Sons Publications. (**l**) Barbed nanofibers. Reproduced with permission [72]. Copyright 2012 AlP Publishing Publications.

Beaded nanofibers: Beaded nanofibers [53,54,55] frequently occur during the electrospinning process (Figure 3a–c). Initially deemed a morphological defect, beaded fibers led to the exploration of various methods to prevent or mitigate their occurrence. With advancing research, the unique structure of beaded fibers contributes to their potential across diverse applications. For instance, Luo et al. [56] generated a range of beaded electrospun fibers. Culturing rat bone marrow mesenchymal stem cells (rBMSCs) on these fibers, they studied the impact of surface nanoroughness on cellular behavior and in vivo subcutaneous bone formation.

Core–shell nanofibers: Core–shell nanofibers (Figure 3d) consist of two or more distinct materials, with one acting as the core and the others enveloping it as the shell, creating a composite with a distinct interface and unique attributes [57]. The coaxial electrospinning method is widely utilized for fabricating core–shell structured fibers. Employing multiple coaxially aligned spinnerets, the components of the spinning liquid are extruded, yielding nanofibers with core–shell structures via the electric field’s force. Coaxial electrospinning and its derivative technologies enable the production of fibers with specialized structures, including hollow fibers [58] (Figure 3e), nanowire–microtubule [59] (Figure 3f), and multi-channel microtubule structures [60] (Figure 3g). The structural diversity and functionality of core–shell fibers facilitate their broad application in the biomedical field. For instance, Han et al. [61] developed core–shell structured drug-loading nanofiber dressings for treatment of deep burns. The core–shell structure facilitated sustained curcumin release, offering antibacterial and anti-inflammatory properties for wound healing. Moreover, the dressing demonstrated significant wound healing efficacy in the Sprague Dawley (SD) rat burn model.

Ribbon Fibers: Ribbon fibers (Figure 3h), a category of electrospun fibers with non-circular cross-sections, exemplify innovation in morphological diversity [62]. Ribbon fiber formation depends on the development of a unique skin structure on the surface of the polymer jet stream during electrospinning; this structure collapses upon solvent evaporation, yielding fibers with a distinct cross-sectional shape. The morphology and structure of ribbon fibers add a new dimension to the diversity of electrospun fiber shapes, enabling design and customization to meet specific application needs [63,64].

Porous Fibers: Porous fibers (Figure 3i) are characterized by their structure, which includes pores on the surface and within the fiber matrix. This enhances the surface area of the fiber, improving functionality across diverse applications [65]. Porous fiber production commonly utilizes phase separation within the electrospinning solution, involving polymer–solvent, polymer–nonsolvent, and polymer–polymer interactions. Porous fibers hold promise for biomedical applications, including facilitating cell attachment and growth for tissue engineering and serving as carriers for controlled drug release [66,67].

Others: Beyond the diverse special-structured fibers previously mentioned, electrospinning enables the creation of additional intriguing nanofibers. These include nanohybrids that emulate natural skeletal structures [68] (Figure 3j), helical fibers [69] (Figure 3k), pleated polystyrene patterns produced via electrospinning [70], high-frequency branched barbed nanowires [71], and inorganic ‘barbed’ nanofibers derived from calcined composite nanofibers [72] (Figure 3l). These novel structures underscore the adaptability of electrospinning in tailoring fiber designs and highlight significant potential in areas like tissue engineering, drug delivery, sensor technology, and biomedical devices, attributable to their distinctive physicochemical characteristics.

#### 2.2.2. Structural Diversity of Nanofiber Membranes

**Figure 4:**
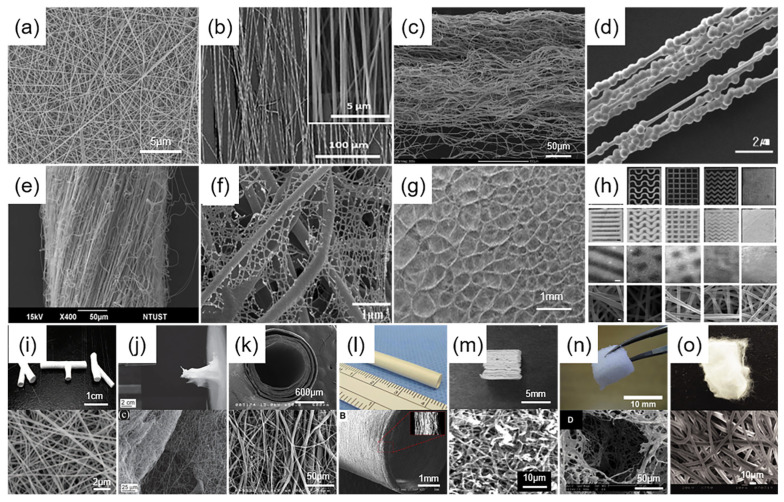
Structural diversity of nanofiber arrangements. (**a**) Random fibers. Reproduced with permission [75]. Copyright 2009 Elsevier Publications. (**b**) Oriented fibers. Reproduced with permission [94]. Copyright 2023 Elsevier Publications. (**c**) Oriented self-curling fibers. Reproduced with permission [80]. Copyright 2010 American Chemical Society Publications. (**d**) Oriented beaded fibers. Reproduced with permission [81]. Copyright 2006 American Chemical Society Publications. (**e**) Nanofiber yarn [83]; (**f**) Spider-web-patterned fibers. Reproduced with permission [87]. Copyright 2023 Elsevier Publications. (**g**) Honeycomb-patterned fibers. Reproduced with permission [88]. Copyright 2019 Elsevier Publications. (**h**) Patterned fibers. Reproduced with permission [89]. Copyright 2021 American Chemical Society Publications. (**i**) Cross-tube fibers. Reproduced with permission [95]. Copyright 2008 American Chemical Society Publications. (**j**) 3D structural fibers. Reproduced with permission [96]. Copyright 2012 John Wiley and Sons Publications. (**k**) Tubular fibers prepared manually. Reproduced with permission [97]. Copyright 2008 John Wiley and Sons Publications. (**l**) Tubular fibers. Reproduced with permission [98]. Copyright 2015 Elsevier Publications. (**m**) 3D fiber stacks. Reproduced with permission [99]. Copyright 2011 John Wiley and Sons Publications. (**n**) 3D fiber scaffolds. Reproduced with permission [100]. Copyright 2008 Elsevier Publications. (**o**) Expanded 3D fiber scaffolds. Reproduced with permission [101]. Copyright 2010 John Wiley and Sons Publications.

Random fibers: Random fibers, as depicted in Figure 4a, represent the most prevalent type of electrospun fibers [75]. They are commonly produced using a standard electrospinning collector, where the charged polymer jet exhibits a natural helical path, culminating in the formation of a continuous, randomly oriented mesh of fine fibers upon the grounded collector plate [76].

Ordered fibers: To produce well-ordered electrospun nanofibers [77] as shown in Figure 4b, researchers have devised multiple techniques such as utilizing mechanical forces from rotating mandrels, electrostatic forces from parallel metal plates, force fields from centrally pinned metal ring or bead arrays, and magnetic fields from permanent magnets [78]. Furthermore, the near-field electrospinning technique [79] has emerged as an effective method for the precise manipulation of fiber alignment, mitigating jet meandering and enhancing polymer jet guidance. In certain instances, these oriented fibers possess supplementary structures [80,81], as depicted in Figure 4c,d. The implementation of these methods enables the fibers, when collected in designated areas, to display a structured organization, presenting significant potential for the innovation of biomedical devices and systems [82].

Nanofiber yarns: Nanofiber yarns [83], depicted in Figure 4e, are three-dimensional fibers with unique structures produced through electrospinning. They are typically created with a water bath collector [84] or a rotating drum [85], utilizing water flow to automatically stretch and form the fibers into continuous yarns. The yarns exhibit significant potential in biomedical applications owing to their strength and flexibility, emulating the structural attributes of the extracellular matrix and offering support and orientation for cellular processes [86]. Despite challenges relating to fiber consistency and production scaling, production of nanofiber yarn is advancing, with ongoing refinements in collection and stretching techniques, promising the development of innovative high-performance biomaterials.

Patterned fibers: Patterned electrospun fiber mats [87,88], as illustrated in Figure 3f–h, have been produced using conductive template collectors and meticulous electric field force manipulation, ensuring a regular fiber arrangement and the formation of defined patterns. Furthermore, the localized nanofiber dissolution method and the low-voltage near-field electrospinning technique have improved fiber patterning capabilities, facilitating high-resolution and tailored designs. These fiber mats, characterized by their precise microstructures, have demonstrated significant effects on enhancing cell behavior and tissue regeneration [89]. The patterned fibers exhibit substantial potential across various applications, including controlled drug delivery, biosensing, and diagnostic technologies [90].

3D nanofibrous macrostructures: According to Sun et al.’s [91] review, four primary strategies are utilized for crafting 3D electrospun fiber macrostructures: prolonged spinning time (constructing thicker fiber structures by increasing the spinning time); post-processing assembly of 2D fiber mats (including folding, layer-stacked electrospinning, sintering, mechanical extension, etc.); direct assembly of the auxiliary factors (using 3D templates or liquid collectors and other auxiliary tools to directly construct 3D structures); self-assembly (using the self-assembly ability of electrospun fibers to form 3D structures through physical forces such as surface tension and electrostatic repulsion). Three-dimensional structures as shown in Figure 4i–o offer cells an additional spatial dimension over two-dimensional fiber mats. These 3D fiber mats facilitate intercellular migration and induce morphological changes [92], processes vital for cell cycle regulation and tissue function [93].

## 3. Application of Electrospun Nanofibers in Wearable Sensors

The human body and its surrounding microenvironment continuously emit a variety of signals, including electrical, mechanical, thermal, humidity, and biochemical, which are crucial for assessing overall health. Advancements in technology have enabled the development of non-invasive wearable sensors capable of detecting signals like pressure, temperature, humidity, gases, biochemical molecules, and light [102]. Electrospun nanofiber membranes possess excellent properties such as breathability, biocompatibility, flexibility, comfort, a high specific surface area, low cost, and ease of mass production. Consequently, they hold broad application potential in the field of wearable sensors for detection of human physiological signals. Sensors can be classified as physical or biosensors, based on the type of signals they detect.

### 3.1. Physical Sensors

**Figure 5:**
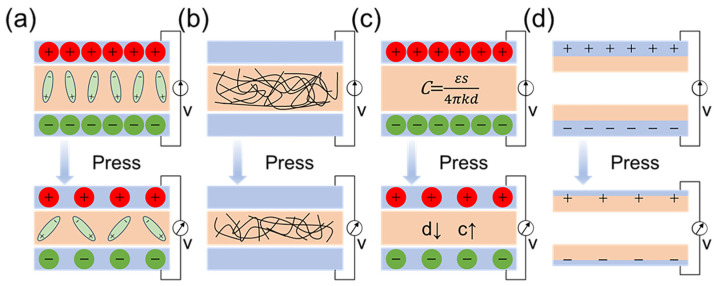
Physical sensors with different operating principles: (**a**) piezoelectric; (**b**) piezoresistive; (**c**) capacitive; (**d**) triboelectric. Reproduced with permission [103]. Copyright 2023 American Chemical Society Publications.

Physical sensors detect physical stimuli, such as temperature, sound, and pressure, converting them into electrical signals for use in measurement and control systems. In human health monitoring, physical sensors are crucial for detecting vital physiological indicators including electrical signals, pulse, physical activity, and body temperature. They convert physical signals to electronic ones in real time via mechanisms like the piezoelectric effect, the triboelectric effect, resistance variation, and capacitance variation [103], as depicted in Figure 5. They typically respond to material deformations like stretching, twisting, bending, or expanding [5]. Hence, physical sensors must exhibit excellent elasticity, durability, high sensitivity, and rapid response times. Notably, electrospun nanofibers are an ideal material for physical sensors due to properties like their low Young’s modulus, high specific surface area, and porous network structure, along with the potential for functionalization through material doping [104].

#### 3.1.1. Monitoring of Electrophysiological Signals

Human electrophysiological signals, such as those detectable via electrocardiography (ECG), surface electromyography (sEMG), or electroencephalography (EEG), are spontaneously emitted during routine activities and serve as reliable health status indicators, in relation to aspects including heart rhythm, muscle contraction, and brain neural function. Thus, real-time monitoring of these physiological signals is essential for the early detection and treatment of diseases [105]. Ultrasonic modification and thermal fusion, when combined with electrospinning, can uniformly disperse nanomaterials, such as metal nanoparticles, throughout nanofibers. This composite approach enhances nanofiber functionality and enables the fabrication of physical sensors with greater precision, significantly boosting the accuracy of physiological electrical signal monitoring [103].

**Figure 6:**
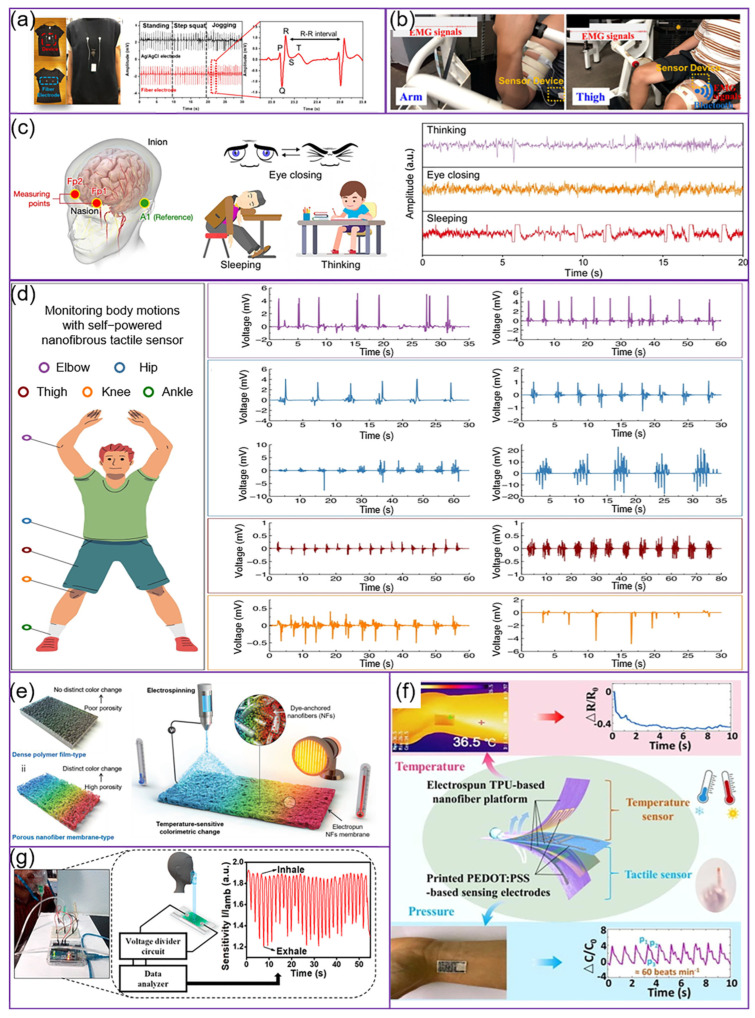
Application of electrospun nanofiber-based physical sensors for human health monitoring. (**a**) ECG monitoring. Reproduced with permission [107]. Copyright 2023 Springer Nature Publications. (**b**) EMG monitoring. Reproduced with permission [110]. Copyright 2023 Springer Nature Publications. (**c**) EEG monitoring. Reproduced with permission [113]. Copyright 2022 American Chemical Society Publications. (**d**) Human motion monitoring. Reproduced with permission [114]. Copyright 2023 Springer Nature Publications. Temperature monitoring: (**e**) thermotropic temperature sensor. Reproduced with permission [115]. Copyright 2022 John Wiley and Sons Publications. (**f**) Resistive temperature sensor. Reproduced with permission [116]. Copyright 2023 Elsevier Publications. (**g**) Respiratory humidity monitoring. Reproduced with permission [117]. Copyright 2019 American Chemical Society Publications.

ECG monitoring: ECG, a visual record of the heart’s electrical activity, is fundamental to diagnosing cardiovascular diseases, assessing cardiac function, and developing treatment plans. Accurately capturing changes in the heart’s electrical signals is invaluable for diagnosing conditions like arrhythmia, myocardial ischemia, and infarction, and it is essential for monitoring cardiac health and adjusting treatment protocols [106]. Li et al. [107] created a wearable smart electronic device by electrospinning a thermoplastic polyurethane nanofiber membrane and coating it with a silver nanoparticle/carbon-based paste to form electrodes (Figure 6a). These fiber-based electrodes exhibited superior tensile strength, electrical conductivity, sensitivity to physiological signals, self-healing capabilities, and sweat resistance, making them suitable for real-time ECG signal collection and health monitoring. Lu et al. [108] developed a flexible, ultra-lightweight, highly conductive multifunctional sensor for ECG signal detection through electrospinning of a PA66 solution, enhanced by ultrasonic modification. This may provide an excellent alternative with a comfortable feeling when worn and accurate detection.

sEMG monitoring: EMG, which directly records neuromuscular electrical activity, is central to diagnosing peripheral nervous system disorders. EMG detects nerve and muscle signal conduction, precisely identifying the location and extent of nerve damage, which is crucial for formulating treatment plans and assessing prognosis. Furthermore, EMG can uncover subclinical abnormalities, aiding in the early detection and treatment of diseases, and it is thus essential in neurological evaluations [109]. Chiu et al. [110] developed AgNP/GO nanofiber electrodes (Figure 6b), which, after 5000 cycles of 50% tensile fatigue tests, retained a stable microstructure, demonstrating potential for long-term ECG and EMG monitoring surpassing conventional electrodes. Huang et al. [111] created a stretchable, flexible nanofiber membrane electrode via electrospinning, successfully implementing it in smart garments for ECG and EMG monitoring. This electrode boasts high conductivity, mechanical durability, and a hydrophobic surface, offering superior skin contact and longevity in comparison with traditional wet electrodes. It is ideal for extended bio-signal recording and can be seamlessly integrated into wearable health monitoring systems.

EEG monitoring: EEG, reflecting the electrical activity of the cerebral cortex, is pivotal for diagnosing neurological disorders, notably epilepsy and other seizure conditions. It captures abnormal brain signals, offering a scientific basis for disease assessment and brain functionality, and it is crucial in determining brain death [112]. Dong et al. [113] created a multifunctional metamaterial fabric with superelasticity and high breathability through electrospinning and hot melting, integrating liquid metal for stable electrical conductivity (Figure 6c). By documenting three typical mental states of volunteers (thinking, eye closing, sleeping), the performance of the material as an EEG electrode was assessed. The results indicated that when this fabric was applied as a skin sensor, its sensitivity to human physiological signals (ECG, sEMG, and EEG) was comparable to that of commercial gel electrodes. Peng et al. [105] developed an e-textile conforming to the skin, capable of monitoring high-fidelity electrophysiological signals (ECG, sEMG, and EEG) as a bioelectrode; the material is effective even underwater, offering a promising zero-energy alternative for health-monitoring electronic skins.

#### 3.1.2. Monitoring of Human Movement

Monitoring human movement is crucial in medical applications. It is essential to modern medical treatment, providing real-time physiological and movement data that inform health assessments, treatment strategies, injury prevention, rehabilitation, and research.

Recently, electrospun nanofiber-based sensors have advanced rapidly. They are capable of capturing abnormal movement signals and monitoring activities like speech, chewing, and swallowing [118]. For instance, Li et al. [114] developed a self-powered, flexible, and breathable tactile sensor from nanofiber membranes (Figure 6d) by electrospinning polyvinylidene fluoride (PVDF) nanofibers and incorporating multi-walled carbon nanotubes (CNTs) and barium titanate (BTO). This sensor can efficiently monitor and recognize motion across various body joints, including elbows, hips, thighs, knees, and ankles. Li et al. [119] developed a flexible piezoresistive pressure sensor utilizing thermoplastic polyurethane (TPU) and electrospun fiber networks. This sensor boasts high sensitivity, a broad operating range, low hysteresis, and structural stability, enabling it to discern various frequencies and pressures for monitoring finger and wrist movements, drinking, and subtle pulse signals. Ahmed et al. [120] developed a highly sensitive piezoresistive strain sensor, which was successfully applied to various body parts such as fingers, fists, elbows, knees, and ankles for motion detection and heart rate monitoring. Mounting it on the glottal node enabled speech recognition, with a remarkable response to articulation, breathing, and swallowing. Cheng et al. [121] reported a high-performance piezoresistive flexible sensor featuring a micro–nano 3D network structure, offering excellent sensitivity, rapid response, a low detection limit, and stability. These properties enabled the sensor to monitor subtle physiological signals, including respiratory intensity, pulse rate, and facial expression changes.

#### 3.1.3. Monitoring of Temperature

Temperature monitoring is a critical medical tool for evaluating an individual’s health status. It serves as a key indicator for early diagnosis, disease monitoring, and assessing treatment efficacy. Minor variations in body temperature may indicate infection, inflammation, or other pathological conditions, playing a crucial role in clinical decision making. Advances in monitoring technology have enabled real-time temperature tracking, which bolsters personalized medicine and public health safety, facilitating more efficient and prompt disease management [122].

Wearable electrospun temperature sensors fall primarily into two categories: thermochromic and resistive types [102]. Kim et al. [115] developed a highly sensitive nanofiber sensor membrane via electrospinning (Figure 6e). This nanofiber membrane, embedded with thermochromic dyes, exhibits greater thermochromic sensitivity and superior light transmittance compared with conventional dense membrane sensors, and is adaptable for wearable devices like masks, patches, and bracelets for precise real-time body temperature monitoring. Wang et al. [116] developed an ultrathin, flexible bifunctional sensor with high sensitivity to both pressure and temperature (Figure 6f). This sensor is also humidity-insensitive, waterproof, and breathable, ensuring comfort during extended wear and showing significant potential in wearable health monitoring.

#### 3.1.4. Monitoring of Respiratory Humidity

Monitoring respiratory humidity is crucial for assessing respiratory health and essential in the early diagnosis and treatment of respiratory diseases. It aids in real-time airway hydration monitoring, optimizes medication delivery, assesses environmental adaptation, prevents infections, and manages hydration during exercise and geriatric care, also assisting in diagnosing sleep apnea [123].

Electrospinning offers significant advantages in fabricating highly sensitive respiratory humidity sensors, such as low cost, ease of operation, and tunability. Iyengar et al. [117] developed a wearable humidity sensor utilizing electrospun PVDF/rGO nanofibers (Figure 6g). This sensor boasts high sensitivity, a rapid response time (~1 s), a comprehensive response range (0–95% RH), low operating voltage, and a streamlined circuit design, enabling accurate detection of nasal breathing and humidity levels, and is suitable for integration into wearable electronics. Feng et al. [124] developed a fiber-optic humidity sensor incorporating hybrid nanofibers. The electrospun SnO_2_/polyvinyl alcohol (PVA) nanofiber membrane enhances the humidity sensitivity of the sensor, achieving peak intensity sensitivity of 0.43 dB/%RH within the 35–75% RH range. Additionally, the sensor features an ultra-fast response time (67 ms) and recovery time (83 ms), suitable for real-time monitoring of human respiration across various rhythms.

### 3.2. Biosensors

Biosensors operate on the principle of interacting with a specific target analyte through physicochemical reactions, generating changes that are then converted into detectable signals. Biosensors can analyze health by detecting and binding specific molecules in body fluids such as saliva, tears, interstitial fluids, sweat, and blood, using attached bioreceptors. Consequently, the quantity and distribution of bioreceptors are critical to biosensor performance. Electrospun nanofibers, with their large specific surface area and high porosity, facilitate the functionalization and diffusion of target molecules, enhancing recognition and sensitivity. Thus, electrospun nanofibers offer significant advantages for biosensor applications [5].

#### 3.2.1. Monitoring of Glucose

Monitoring glucose levels in body fluids is crucial for medical diagnosis and treatment. It is vital for early diagnosis, treatment monitoring [125], and efficacy evaluation of diabetes mellitus, as well as for preventing and managing associated cardiovascular issues, neuropathy, and renal disease. Thus, precise glucose monitoring in body fluids is indispensable for maintaining health, optimizing treatment plans, and enhancing quality of life.

**Figure 7:**
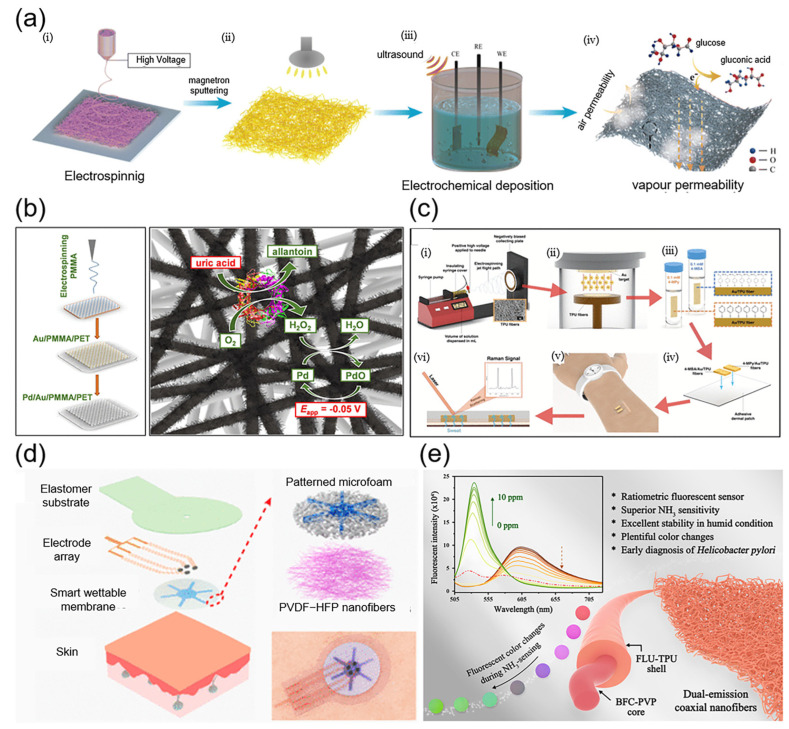
Application of biosensors based on electrospun nanofibers for human health monitoring. (**a**) Preparation process of nanofiber sensors for glucose monitoring. Reproduced with permission [128]. Copyright 2023 Royal Society of Chemistry Publications. (**b**) Schematic of the preparation of nanofiber sensors for uric acid monitoring. Reproduced with permission [131]. Copyright 2024 American Chemical Society Publications. (**c**) Nanofiber sensors for pH monitoring. Reproduced with permission [135]. Copyright 2021 American Chemical Society Publications. (**d**) Structural schematic of nanofiber sensors for inorganic monitoring. Reproduced with permission [140]. Copyright 2024 American Chemical Society Publications. (**e**) Nanofiber sensors for human respiratory gas monitoring. Reproduced with permission [146]. Copyright 2022 Elsevier Publications.

Glucose sensors are classified into enzymatic and non-enzymatic types [126]. For instance, Puttananjegowda et al. [127] developed an electrospun nanofiber membrane from silicon carbide nanoparticles (SiCNPs) and conductive polymer (CP), for use in electrochemical enzyme-based glucose sensors. The nanostructure of the material improved glucose oxidase (GOx) binding, allowing glucose detection in a 5 mM potassium permanganate solution at +0.6 V. Luo et al. [128] introduced a novel non-enzymatic electrochemical glucose sensing patch utilizing electrospun polyurethane (PU) fiber mats (Figure 7a). The patch incorporates well-dispersed platinum nanopine needles via magnetron sputtering and ultrasound-assisted electrodeposition, resulting in high electrical conductivity, stretchability, sensitivity, and a low detection limit. The patch enables non-enzymatic catalytic glucose oxidation under neutral conditions, offering a solution for wearable glucose monitoring that is easily fabricated and boasts superior performance.

#### 3.2.2. Monitoring of Uric Acid

Elevated uric acid levels, an important biomarker, may signal purine metabolism disorders or renal excretion issues [129]. Regular uric acid level monitoring enables doctors to adjust treatments promptly, manage hyperuricemia effectively, and prevent joint inflammation and kidney stones associated with uric acid crystallization. Monitoring and proper treatment can significantly reduce the risk of these health issues [130].

Uric acid sensors made using electrospinning leverage the high specific surface area of nanofibers to immobilize uric acid enzymes, enhancing sensitivity and detection accuracy by increasing contact with uric acid molecules. Leote et al. [131] developed a flexible uric acid (UA) biosensor utilizing palladium-coated submicron electrospun poly(methyl methacrylate) (PMMA) fibers (Figure 7b). Metallizing gold and immobilizing uricase (UrOx) on a PET substrate resulted in high sensitivity and a low detection limit (12 μM) for UA. The biosensor demonstrated recoveries of 98–105% for UA detection in body fluids and retained 78% of its initial sensitivity over three months. Wei et al. [132] described a flexible sweat biosensor employing electrospun carbon nanofibers for uric acid detection. Thanks to its abundant active sites, oriented graphitized layers, and inherent shape adaptability, the sensor offers ample access to uric acid molecules, ensuring efficient electron transfer. This sensor accurately and selectively measured uric acid levels in artificial sweat, offering a promising strategy for wearable biosensor development and promoting the adoption of personalized diagnostic tools.

#### 3.2.3. Monitoring of pH

Monitoring the pH of body fluids is crucial for evaluating acid–base balance, metabolic processes, and respiratory health. This provides vital data for diagnosing and treating diseases [133], enabling physicians to promptly adjust treatment plans for optimal patient care. Additionally, pH monitoring is crucial for assessing treatment efficacy and tracking disease progression, aiding clinical decision making and enhancing care quality [134]. Incorporating pH-responsive molecules during electrospinning enables the creation of sensors that can detect pH levels in body fluids. Chung et al. [135] developed a flexible, nanofiber-structured surface-enhanced Raman scattering (SERS)-active substrate by integrating electrospun thermoplastic polyurethane (TPU) with Au sputter coating (Figure 7c). The SERS substrate, functionalized with pH-responsive molecules 4-mercaptobenzoic acid (4-MBA) and 4-mercaptopyridine (4-Mpy), is suitable for wearable sweat pH sensing. The SERS pH sensor offers high resolution, rapid sweat absorption, excellent repeatability, and reversibility, with stable performance maintained over 35 days. Ha et al. [136] introduced a wearable colorimetric sweat pH sensor based on curcumin and thermoplastic polyurethane (C-TPU) electrospun fibers. The color of the C-TPU fibers changes from yellow to red when sweat pH increases. Responding to pH changes with color shifts, this sensor enables rapid and sensitive sweat pH detection, aiding in the development of smart diagnostic garments for conditions like cystic fibrosis that necessitate ongoing sweat pH monitoring.

#### 3.2.4. Monitoring of Inorganic Substances

Monitoring ion concentrations in body fluids is crucial for assessing electrolyte balance, neuromuscular function, and acid–base status [137]. Abnormal ion level fluctuations are indicative of pathological conditions like kidney disease, cardiovascular disease, and metabolic disorders [138]. Electrospun nanofibers, developed as sweat-absorbing materials, can capture and analyze inorganic components of sweat, offering an effective tool for health monitoring. Lopresti et al. [139] developed a green wearable sensor for sweat absorption and chloride ion detection. Calibration tests confirmed the sensor’s ability to sensitively and reproducibly measure chloride ions in sweat samples from healthy volunteers. The results supported the feasibility of applying this green sweat sensor directly to human skin for chloride ion quantification. Sun et al. [140] proposed a stretchable smart patch for in situ multiple sweat fractionations (Figure 7d). The patch, combining a microporous membrane with a nanofiber layer, selectively extracts and directs the flow of skin sweat. The integrated electrochemical sensor array can measure multiple biomarkers, including pH, K^+^, and Na^+^, simultaneously. These in situ measured concentrations are consistent with those obtained through postanalysis using standard lab techniques.

#### 3.2.5. Monitoring of Gases

Specific breath biomarkers like dimethyl sulfide, acetone, ammonia, and toluene are linked to conditions such as bad breath, diabetes, kidney disease, and lung cancer, respectively [141]. Trace amounts of these markers in exhaled breath can indicate underlying diseases. Hence, developing sensors to detect these biomarkers is essential for medical diagnostics [142,143,144]. Electrospinning can produce nanofibrous materials with core–shell structures, and optimizing these structures can significantly improve gas sensor performance. Fu et al. [145] developed a Co_3_O_4_/TiO_2_ core–shell nanofiber gas sensor by using coaxial electrospinning. The sensor can detect acetone at levels as low as 500 ppb, potentially aiding in the noninvasive clinical detection of acetone in diabetic patients’ exhaled breath. Also using coaxial electrospinning, Cai et al. [146] developed a ratiometric fluorescent nanofiber sensor for NH_3_ detection in exhaled breath (Figure 7e). This sensor demonstrated high sensitivity to NH_3_ (with a theoretical detection limit of 7.16 ppb), a rapid response time of approximately 2.2 s, and excellent stability, particularly in high-humidity environments.

## 4. Application of Electrospun Nanofibers in Personal Protective Equipment

The protection of healthcare workers is a primary concern in patient care and during exposure to infectious diseases. The global use of personal protective equipment (PPE) is recognized as a key strategy for reducing pathogen transmission between patients and healthcare workers (HCWs). PPE also prevents exposure to substances like body fluids (e.g., blood, urine, vomit), medications, and feces, in addition to microorganisms. PPE types can be categorized into body protection, face protection, and hand protection [12].

### 4.1. Masks

Electrospun nanofibers in masks provide numerous advantages: efficient particulate filtration, potent antibacterial and antiviral capabilities, superior breathability and comfort [147], enhanced mechanical strength and durability, and multifunctionality through designs like moisture regulation, electromagnetic shielding, and self-cleaning. Additionally, using biodegradable materials in electrospinning supports environmental protection and minimizes pollution. These advantages position electrospun masks as offering superior personal protection, comfort, and health and safety for users [148]. The applications of electrospun nanofiber membranes in masks are categorized into four primary types: durable masks, antimicrobial masks, filtration masks, and biodegradable masks.

#### 4.1.1. Durable Masks

**Figure 8:**
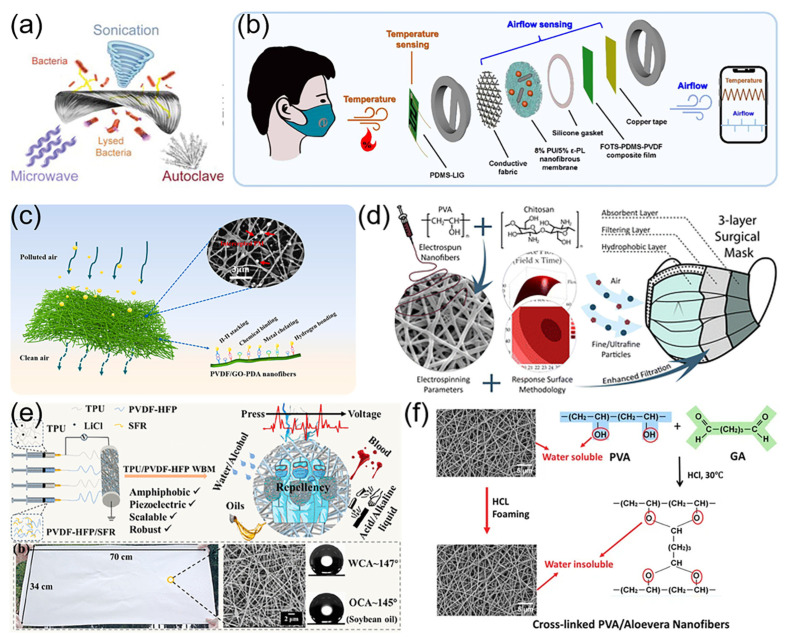
Application of electrospun nanofibers in personal protective equipment. (**a**) Durable mask. Reproduced with permission [150]. Copyright 2022 John Wiley and Sons Publications. (**b**) Antimicrobial mask. Reproduced with permission [153]. Copyright 2024 IOP Publishing Publications. (**c**) Filtering mask. Reproduced with permission [154]. Copyright 2023 Elsevier Publications. (**d**) Preparation process of biodegradable mask. Reproduced with permission [157]. Copyright 2023 Royal Society of Chemistry Publications. (**e**) Preparation process of protective apparel. Reproduced with permission [163]. Copyright 2024 American Chemical Society Publications. (**f**) Antimicrobial nanofibers that can be used to prepare gloves [164].

The primary filtration mechanisms of nanofiber membrane masks—diffusion and direct interception—are attributed to the nanoscale fiber diameter, which allows reuse after disinfection and decontamination. For instance, Mamun et al. [149] investigated the reusability of electrospun nanofiber mats. They washed the mats at various temperatures and assessed their air permeability and moisture resistance. The results indicated no significant loss in air permeability post-washing, confirming the potential for electrospun nanofiber masks to be reused. This offers a safe and effective method for extending the life cycle of personal protective equipment, particularly sanitary masks. Le et al. [150] developed an innovative, reusable, self-sustaining, highly efficient, and moisture-resistant air filtration membrane for masks (Figure 8a). The membrane demonstrated robust stability and moisture resistance even under wet conditions and could be sterilized for reuse using standard methods like ultrasonication, autoclaving, or microwave treatment.

#### 4.1.2. Antimicrobial Masks

Antimicrobial fabrics hold significant potential for practical applications, including personal protective equipment, healthcare, and disease prevention [151]. Researchers have utilized electrospinning technology to develop a range of highly effective antimicrobial masks. For instance, Bansode et al. [152] developed antimicrobial masks by electrospinning copper oxide (CuO)-based nanocomposites with varying concentrations of polyacrylonitrile (PAN) and curcumin. Characterization tests confirmed that the PAN/CuO/curcumin nanofiber-based materials exhibited antibacterial efficacy. Lan et al. [153] introduced a smart mask that integrated airflow and temperature sensing (Figure 8b), high-efficiency filtration, and superior antimicrobial properties. The nanofiber membrane served multiple roles: as a filter, antimicrobial layer, and triboelectric material, demonstrating outstanding filtration and antimicrobial capabilities.

#### 4.1.3. Filtration Masks

Electrospinning technology can produce nanofiber filter membranes that offer high filtration efficiency, low air resistance, excellent mechanical properties, and customizable pore structures and surface chemistries. Consequently, electrospun nanofiber membranes can effectively capture tiny particles with high efficiency and durability, offering significant advantages for filtration mask applications. Liu et al. [154] developed a high-efficiency mask filter material through blend electrospinning and in situ surface polymerization (Figure 8c). The material demonstrated a high removal efficiency of 99.37% for 0.3 μm particulate matter (PM0.3) particles as well as low air resistance of 56 Pa, maintaining 98.52% interception efficiency post-destaticization and retaining over 92.00% PM0.3 filtration efficacy after 72 h of use. Shao et al. [155] created cellulose–silica nanofiber (C-SNF) aerogels embedded with zeolite imidazolium framework-67 (ZIF-67) for filtration masks, using electrospinning and freeze drying. The C-SNF aerogel provided a filtration efficiency of 99.91% for 0.3 μm salt particles, sustained 99.92% removal efficiency for PM2.5, and achieved a 93.75% removal rate for formaldehyde.

#### 4.1.4. Biodegradable Masks

Traditional masks, which are not degradable, are typically disposed of through incineration or landfilling, leading to environmental pollution. Electrospinning technology can produce degradable nanofiber masks, mitigating environmental pollution [156]. Mata et al. [157] created biodegradable nanofibers via electrospinning, employing PVA and chitosan (CS) as alternatives to conventional polymers in commercial surgical masks (CSMs) (Figure 8d). The optimized samples demonstrated filtration efficiencies on a par with N95 masks and adhered to international standards. The nanofiber membrane was more effective at capturing nanoscale particles, including airborne viruses and fine/ultrafine pollutants, compared with CSMs. Yang et al. [158] developed a biodegradable multiscale fiber filter, comprising polylactic acid (PLA) micron fibers and bacterial cellulose (BC) nanofibers. The filter showcased over 99.89% PM filtration efficiency with a low pressure drop (104 Pa) and sustained over 99.68% PM removal even after prolonged use under 90% relative humidity. The successful development of this material offers significant insights for the creation of biodegradable, high-efficiency protective air filters.

### 4.2. Protective Suits

Electrospun nanofibers, due to their high specific surface area and porosity, offer superior filtration and breathability in medical protective garments. Additionally, customized designs can integrate multifunctional properties such as antimicrobial [159], flame-retardant, and electromagnetic shielding capabilities, enhancing the suits’ durability and comfort [160]. This offers an efficient, scientific approach to personal protection in medical settings. For instance, Aijaz et al. [161] developed an innovative protective garment using a bio-based electrospun nanofiber membrane, with waterproof, antimicrobial, breathable, and cooling properties and high tensile strength. This material is suitable for applications such as adult diapers, outdoor wear, and assistive devices for individuals with disabilities, potentially enhancing the quality of life for the disabled and elderly. Zhang et al. [162] created a fluorine-free, multifunctional nanofiber membrane using electrospinning. By precisely controlling the UV531 concentration, the membrane offers excellent water repellency, breathability, mechanical strength, and UV protection up to 238.23 UPF, along with superior acid and alkali resistance. These attributes render it an eco-friendly and versatile option for outdoor protection, extreme environment applications, and military gear. Xing et al. [163] developed a TPU/poly(vinylidene fluoride-co-hexafluoropropylene) (PVDF-HFP) nanofiber membrane with an interwoven fiber structure, through multi-needle electrospinning (Figure 8e). The membrane offers excellent waterproofing, withstanding water pressures up to 108 kPa, along with high air and water vapor permeability. Moreover, its superior mechanical properties and ability to monitor water pressure via voltage measurement add to its functionality. These characteristics suggest a broad range of potential applications for the nanofiber membrane in protective apparel and wearable electronics.

### 4.3. Gloves

Electrospinning nanofiber technology has not yet been extensively applied in the production of gloves for personal protective equipment (PPE). Khanzada et al. [164] successfully developed aloe vera (AV)/PVA nanofibers embedded with aloe vera extract, using the electrospinning technique (Figure 8f). They adjusted the aloe vera concentration between 0.5% and 3% to produce four nanofiber samples with varying AV content. The results demonstrated significant antimicrobial activity of these nanofibers against *Staphylococcus aureus* and *Escherichia coli*, with particular efficacy against *S. aureus*. These findings suggest that AV/PVA nanofibers hold substantial potential for developing PPE, including medical protective clothing and gloves, potentially enhancing the safety and protection of healthcare workers.

## 5. Application of Electrospun Nanofibers in Personal Temperature Management

Personal thermal management, encompassing heating, cooling, and thermal acclimatization, is essential for enhancing comfort in daily life. The human body typically finds comfort within a temperature range of 20 °C to 27 °C and a relative humidity range of 35% to 60% [165]. However, extreme weather, climate change, and high-density human activity can lead to heat and sweat production that surpasses the body’s thermal regulation capabilities, resulting in severe thermal stress or potentially fatal illnesses [166]. Electrospun nanofibers, characterized by their ultra-fine microstructure, offer superior breathability and moisture management, thus efficiently regulating temperature for personal thermal management. They are crucial materials for both thermal comfort and energy conservation [167].

### 5.1. Active Thermal Management

Active thermal regulation, a method of thermal management, typically depends on an external energy source or control system, necessitating electricity or other forms of energy.

Electrospinning is a widely adopted and efficient method for producing thin nanofibers and microporous conductive membranes, attributed to its low cost, straightforward process, and customizable structural properties. The successful development of diverse conductive nanofiber membranes for active thermal management has been achieved by incorporating various nano-dopants, including silver nanoparticles, graphene nanosheets, and carbon nanotubes. For instance, Xu et al. [168] utilized electrospinning to create a fiber network electrode embedded with liquid metal particles. The electrode exhibits superior electrical conductivity, mechanical stretchability, and fatigue resistance, making it suitable for use as a breathable, stretchable, and durable Joule heater for long-term wearable warming therapy. Wang et al. [169] developed a wearable, breathable nanofiber-based sensor that excels in thermal management, consisting of micropatterned CNT/TPU nanofiber electrodes, microporous ionic aerogel electrolytes, and microstructured silver/TPU nanofiber electrodes. By applying a current to the bottom resistive electrode, the sensor actively generates Joule heating, rapidly warming to offer on-demand temperature control. This adjustable thermal sensor demonstrates significant potential for monitoring and thermotherapy in cold environments. Yu et al. [170] created a superhydrophobic, highly conductive three-layer fabric using electrospinning and additional techniques. The fabric boasts high electrical conductivity, rapid heating, and energy-efficient asymmetric heating. Experiments indicated that the polyamide/fluorinated polyurethane (PA/FPU) nanofiber layer reached over 83 °C within 90 s at 24 V, whereas the FPU/TiO_2_ layer peaked at 53 °C. This suggests the potential of the fabric for use in the next generation of electrically heated textiles.

### 5.2. Passive Thermal Management

**Figure 9:**
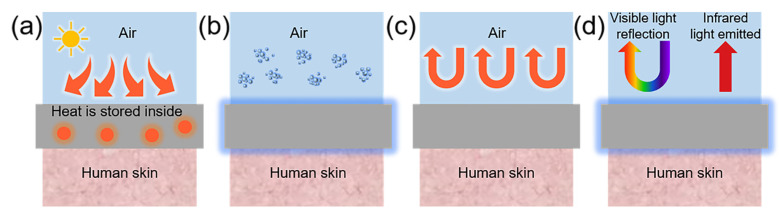
Schematic diagram of the principle of passive thermal management methods. (**a**) Thermal storage; (**b**) evaporative cooling; (**c**) thermal insulation; (**d**) radiative cooling. Reproduced with permission [171]. Copyright 2023 Springer Nature Publications.

Passive thermal regulation is a method of thermal management that does not depend on external energy sources or active control systems, instead utilizing the inherent properties of the material for heat transfer and emission. The use of electrospun nanofiber membranes in passive thermal management encompasses four primary applications: thermal storage, evaporative cooling, thermal insulation, and radiative cooling [171], as illustrated in Figure 9.

#### 5.2.1. Thermal Storage

Phase-change materials (PCMs) can serve as efficient thermal energy storage media, absorbing and releasing energy via latent heat at specific phase-change temperatures [165]. However, blending PCMs with conventional textiles can lead to leakage from the materials due to washing, heating, friction, or abrasion, thus limiting their practical versatility. To address this issue, coaxial electrospinning has been suggested in order to produce fibers with a core–shell structure, encapsulating the PCM (core) within a polymer matrix (shell), thereby offering an efficient encapsulation method for PCMs. Feng et al. [172] utilized coaxial electrospinning to prepare microfibers featuring a PU shell and a polyethylene glycol (PEG) core, creating a core–shell structure boasting high phase-change enthalpy (60.4 J/g), effective encapsulation, thermal energy storage, and robust mechanical properties. The PU/PEG porous membrane demonstrated a thermally activated shape memory effect, enabling macroscopic shape deformation and the adaptive adjustment of microscopic pore sizes upon heating. Furthermore, the porous membrane possessed thermal regulation capabilities and exhibited temperature-sensitive moisture permeability.

#### 5.2.2. Evaporation Cooling

Evaporative cooling is a technology that harnesses the body’s natural mechanisms for regulating excess heat. It achieves cooling by promoting sweat evaporation to absorb body heat, thus maintaining thermal comfort without external energy sources [173]. Utilizing electrospinning technology, researchers have developed two types of evaporatively cooled fabrics. One type is a hygroscopic fiber membrane that rapidly absorb sweat for cooling. For instance, Li et al. [174] created 1D nanofibers and 2D porous fabrics with moisture absorption, durability, ductility, breathability, washability, and antimicrobial properties through airflow-assisted electrospinning. These fabrics enable efficient atmospheric water adsorption, humidity management, evaporative cooling, and body temperature regulation. However, due to limited hygroscopic capacity and a slow evaporation rate, researchers developed Janus nanofiber membranes featuring wettability gradients for unidirectional moisture conduction, thereby optimizing the cooling effect. For example, Lei et al. [175] proposed a wettability-gradient-induced-diode (WGID) membrane, combining PVDF and PU nanofibers with MXene-engineered polyurethane membranes (PU@MXene). This membrane facilitates heat dissipation and hygroscopic transport, enabling water transport without reverse osmosis for passive evaporative cooling.

#### 5.2.3. Thermal Insulation

Thermal insulation is a strategy that maximizes heat transfer blockage using carefully designed thermal barrier materials, offering an efficient method for achieving insulation without external energy consumption. This method effectively reduces heat convection and radiation by creating an efficient thermal barrier around the body, leading to temperature regulation, energy conservation, and increased comfort [167].

Electrospun nanofiber membranes used as an intermediate layer can enhance the thermal insulation properties of materials. For instance, Gu et al. [176] developed a hierarchical nanofiber (HNF) textile to enhance thermal insulation and manage radiant heat, effective for personal heat management in extreme temperatures. The textile is composed of a radiative cooling layer, an intermediate thermal insulation layer, and a radiative heating layer. The intermediate nanofiber membrane has low thermal conductivity, which minimizes heat loss in cold conditions and blocks external heat in hot conditions.

Electrospun nanofibers can also be formulated into aerogels to enhance thermal insulation properties. For example, Wang et al. [177] synthesized an ultra-lightweight, mechanically robust polyimide (PI) aerogel with superior thermal insulation by creating a network of three-dimensionally interlaced coiled nanofibers during electrospinning. The PI aerogel, with its ultra-lightweight nature, extreme temperature tolerance, and thermal insulation, offers an ideal solution for maintaining human thermal comfort in extreme conditions.

#### 5.2.4. Radiative Cooling

Radiant cooling maximizes thermal radiation through the atmospheric window to dissipate heat. It also minimizes the absorption of incident atmospheric radiation, achieving cooling without energy consumption [178]. Electrospun fiber membranes, characterized by high porosity and surface area, are ideal for radiative cooling; numerous studies have explored their application in this field. For instance, Wu et al. [179] developed a mid-infrared spectrally selective hierarchical fabric (SSHF). The fabric exhibits high emissivity in the atmospheric transmission window through its molecular design, minimizes net heat gain, and boasts a solar spectral reflectance of 0.97. When vertically positioned in a simulated urban environment, the fabric was 2.3 °C cooler than a solar reflective broadband emitter. It also offers excellent wearability. Via one-step electrospinning, Cheng et al. [180] created a breathable, leather-like nanotextile (LNT) capable of facilitating efficient daytime radiative cooling and heating.

## 6. Application of Electrospun Nanofibers in Wound Dressings

In clinical practice, wound dressings are crucial for preventing infection and promoting healing [181]. While conventional dressings like gauze, foam, and sponge are widely used for their cost effectiveness, they act as passive barriers, potentially causing wound dehydration and adhesion, which can slow the healing process [182]. In contrast, novel dressings, particularly electrospun nanofiber membranes, offer significant advantages because of their unique structural properties. Their structure resembles the natural extracellular matrix, providing an ideal microenvironment for cell proliferation and differentiation [183,184]. Additionally, their high porosity and large specific surface area facilitate water–air exchange and efficient drug loading, accelerating the wound healing process. These properties suggest that electrospun nanofiber membranes hold great potential for the development of biomedical dressings [185,186].

### 6.1. Hemostasis

**Figure 10:**
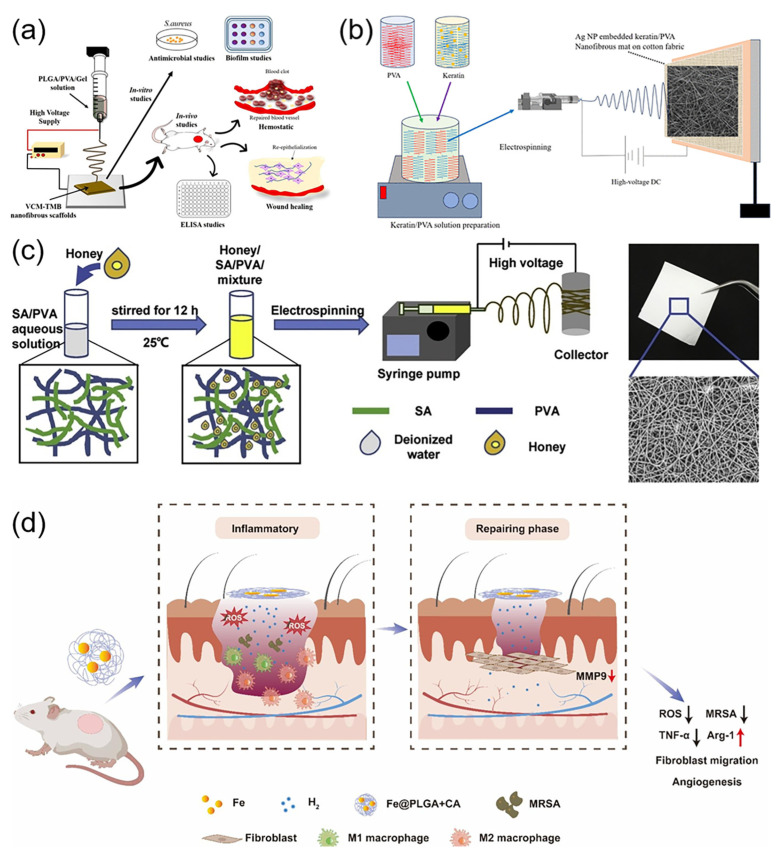
Application of electrospun nanofibers in wound dressings. (**a**) Schematic of the preparation of fibrous membrane containing thrombin. Reproduced with permission [190]. Copyright 2023 Elsevier Publications. (**b**) Schematic of the preparation of antimicrobial fibrous membrane containing silver nanoparticles. Reproduced with permission [193]. Copyright 2024 American Chemical Society Publications. (**c**) Schematic of the preparation of electrospun nanofibers with antimicrobial and antioxidant functions. Reproduced with permission [201]. Copyright 2019 Elsevier Publications. (**d**) Nanofibrous membrane used for pro-angiogenesis. Reproduced with permission [205]. Copyright 2024 Elsevier Publications.

Rapid hemostasis is crucial in wound management and essential for preventing shock and aiding wound healing. Traditional hemostasis methods, like applying direct pressure with gauze, are simple but can lead to issues like additional blood loss and gauze adhesion to the wound, potentially slowing healing and increasing risk of infection. To address these issues, researchers have explored materials with coagulation properties, like chitosan, as well as porous, expandable structures and hemostatic-agent-laden dressings for more effective hemostasis [187]. Li et al. [188] created a nonwoven nanofiber composite using electrospinning, incorporating PVA, kaolin, and insect-derived γ-chitosan. Notably, γ-chitosan from *Protaetia brevitarsis seulensis* exhibited high yield and water-binding capacity. The material showed improved biocompatibility and a substantial hemostatic effect. Furthermore, with a cell survival rate over 86% and a clotting time 2.5 times faster than pure PVA, the material’s potential as an efficient hemostatic dressing is evident. Huang et al. [189] developed a 3D chitosan/poly (vinyl alcohol)-tannic acid (3D-TA) porous nanofiber sponge via electrospinning. This sponge offers significant structural advantages over 2D fiber membranes. Its unique fluffy structure provides the material with enhanced porosity, water absorption, and retention. Most importantly, 3D-TA significantly improves hemostatic performance. Pandey et al. [190] created a multifunctional nanofiber dressing using electrospinning technology (Figure 10a). The dressing, containing thrombin (TMB) and vancomycin (VCM), demonstrated good biocompatibility. In vivo rat model experiments showed that the dressing significantly reduced bleeding time, accelerated coagulation, and promoted wound closure, also lowering inflammatory factor levels. These properties establish a scientific basis for its potential use as an efficient hemostatic and wound healing dressing.

### 6.2. Antimicrobial

Wound infection is critical during the healing process, potentially causing delayed healing, disfigurement, and even death [191]. As an innovative material for wound care, antimicrobial electrospun nanofibers can integrate various antimicrobial agents, including antibiotics, metal nanoparticles, plant extracts, and antimicrobial peptides. These multifunctional materials offer significant antimicrobial effects and excellent biocompatibility, reducing infection risk and accelerating wound healing, thus supporting research and clinical applications in wound management. For instance, using electrospinning, Sun et al. [192] created PCL/silk fibron (SF) nanofiber dressings loaded with N-chloro-N-fluorobenzenesulfonamide (CFBSA). CFBSA, a novel chlorinating agent, showed broad-spectrum antimicrobial and bactericidal effects with low cytotoxicity to eukaryotic cells. The optimized CFBSA-loaded nanofiber dressing demonstrated excellent in vitro antimicrobial activity, with its biocompatibility and antimicrobial efficiency ensured by the appropriate CFBSA concentration. Hassan et al. [193] created composite nanofiber membrane wound dressings containing wool keratin (WK)/PVA and silver nanoparticles (AgNP), via electrospinning on cotton (Figure 10b). The Ag NP-loaded WK/PVA nanofiber membrane showed excellent antimicrobial activity against *Staphylococcus aureus* and *Pseudomonas aeruginosa*, common bacteria causing infections, and was cytocompatible with L-929 mouse fibroblasts. Fahimirad et al. [194] used electrospinning to create a chitosan–nanoparticle composite nanofiber dressing containing the antimicrobial peptide LL37 and vascular endothelial growth factor (VEGF). The dressing showed excellent antibacterial activity against methicillin-resistant Staphylococcus aureus (MRSA). The results of in vitro and in vivo experiments indicate that the dressing is biocompatible and can reduce the inflammatory response.

### 6.3. Antioxidant or Anti-Inflammatory

In the wound healing process, a moderate inflammatory response aids in clearing necrotic tissue and harmful substances, facilitating cell proliferation and tissue repair [195]. However, an excessive inflammatory response can cause macrophages to release excessive cytokines and enzymes, potentially leading to edema, pus formation, and necrosis, which may hinder healing. Reactive oxygen species (ROS) are key in exacerbating the inflammatory response. High levels of ROS, a byproduct of aerobic respiration, can induce oxidative stress and cell damage [196,197,198]. Consequently, researchers have developed antioxidant or anti-inflammatory electrospun nanofibers to mitigate the inflammatory response and enhance wound healing by lowering ROS levels. For instance, Abdelbasset et al. [199] created a wound dressing by blending methoxy phenol (MQ) into an electrospun chitosan/carboxymethyl cellulose (CMC) fiber membrane. The 0.3% MQ dressing demonstrated the highest cellular activity and antioxidant protection. In vitro studies revealed that MQ enhanced the anti-inflammatory and antioxidant properties of the dressing. Using electrospinning, Yuan et al. [200] developed a poly(L-lactide-co-glycolide) (PLGA)/gelatin (PG)-based fiber dressing enriched with pro-biotic peptide (BP) and oregano essential oil (OEO). In vitro experiments indicated the dressing’s anti-inflammatory, antioxidant, and antimicrobial properties, while in vivo studies confirmed its promotion of wound healing, along with anti-adhesion and anti-infection effects, after 14 days of implantation. Due to its antimicrobial, anti-inflammatory, and antioxidant properties, honey has been a staple in wound care since antiquity. Tang et al. [201] electrospun alginate, PVA, and honey into nanofibrous membranes featuring a 3D structure that fostered cell adhesion and proliferation (Figure 10c), along with antimicrobial and antioxidant activities, showcasing its potential as a highly effective wound dressing.

### 6.4. Promoting Cell Proliferation or Angiogenesis

During wound healing, fibroblasts proliferate at the edges and migrate across the wound surface, producing fibrous tissue and matrix to cover the area. New capillaries penetrate the matrix, delivering nutrients essential for skin tissue remodeling and healing. Consequently, cell proliferation, migration, and angiogenesis are crucial for wound healing [202,203]. Researchers have developed various electrospun nanofiber wound dressings to enhance these processes, promoting cell proliferation and angiogenesis. Using electrospinning and 3D printing, Cojocaru et al. [204] created a bicomponent scaffold (BiFp@Ht) for wound dressing applications, co-loaded with a prodrug and a drug. Quantitative in vitro assessments showed that the BiFp@Ht scaffold had good biocompatibility and no cytotoxicity to HeLa cells, and it supported the highest proliferation of HeLa cells on Fp nanofiber membranes. Zhang et al. [205] developed a PLGA electrospun nanofiber membrane enriched with citric acid (CA) and ferric (Fe) nanoparticles (Figure 10d). The membrane continuously generated and released hydrogen via the reaction between Fe and CA in an acidic microenvironment. Hydrogen molecules reduced matrix metalloproteinase 9 levels to improve fibroblast migration, scavenged or neutralized ROS, and modulated the immune response from pro-inflammatory to anti-inflammatory. This promotes angiogenesis, wound healing, and the acceleration of skin regeneration.

## 7. Summary and Future Perspectives

### 7.1. Summary

Electrospun nanofibers, with their high specific surface area, excellent permeability, and tunable surface functionalization, are crucial in the field of non-implantable medical applications. This paper begins by comparing the advantages and disadvantages of the two primary electrospinning techniques based on the spinneret system, and then discusses the structural diversity of nanofibers and their respective application areas. At the core of this paper, we detail the key applications of electrospun nanofibers in non-invasive medical fields, as follows:

1. Wearable smart sensors: This paper discusses the use of nanofibers in smart sensor applications, categorized into physical and biosensors based on the signal types they detect. Physical sensors leverage the mechanical properties of nanofibers to detect physical signals like pressure and deformation, whereas biosensors capitalize on their high specific surface area and biocompatibility for the sensitive detection of biomolecules, enabling the monitoring of physiologically active substances;

2. Personal protective equipment (PPE): This paper provides a detailed overview of nanofiber applications in PPE. Based on the area of protection, PPE is categorized into face protection (masks), body protection (protective clothing), and hand protection (gloves). The high porosity and filtration efficiency of electrospun nanofibers render them well-suited for filtering airborne particles and liquid splashes, thereby enhancing PPE safety and comfort;

3. Temperature management: This paper categorizes temperature regulation applications into active and passive methods, based on their regulatory mechanisms. Active regulation employs smart responsive materials for dynamic temperature control, whereas passive regulation utilizes the insulating and breathable attributes of nanofibers to maintain a stable thermal environment for the human body;

4. Wound dressings: This paper concludes with a summary of nanofiber applications in wound care, highlighting the versatility of nanofiber dressings in wound healing and emphasizing their functional properties including hemostasis, antimicrobial activity, anti-inflammatory effects, antioxidant capabilities, and the promotion of cell proliferation and angiogenesis.

### 7.2. Future Perspectives

Despite its remarkable potential, electrospinning still faces some challenges in its practical applications.

1. Productivity: Conventional single-needle electrospinning equipment has a low production rate that restricts its industrial use. Multi-needle technology enhances efficiency but can lead to electric field interference and non-uniform distribution of fibers;

2. Mechanical properties: The often mechanically weak nature of electrospun nanofiber membranes restricts their use in applications requiring high load bearing or abrasion resistance;

3. Environmental and health impacts: The solvents and additives used in electrospinning can pose risks to the environment and human health, necessitating the development of safer, more eco-friendly spinning solutions;

4. Technical bottlenecks: Clogging of spinning needles is common, particularly with high-viscosity or high-concentration polymer solutions, potentially causing production halts and issues with product uniformity.

In addressing the existing challenges, the future development of electrospinning technology should concentrate on the following key areas:

1. Material modification and innovation, improving nanofibers’ mechanical properties and biocompatibility by integrating material science advancements to address the unique requirements of non-invasive medical applications;

2. Production process innovation, investigating and refining production methods, including multi-needle electrostatic spinning and needle-free electrospinning, to enhance efficiency and minimize costs for large-scale manufacturing;

3. Environmental adaptability research, focusing on developing materials that sustain their performance across various environmental conditions and intensifying research into their environmental adaptability;

4. Interdisciplinary cooperation, encouraging collaboration across material science, biomedicine, engineering, and other fields to address technical challenges collectively;

5. Clinical translation and regulatory standards, including intensifying research into clinical translation and developing robust regulations and standards to ensure the safety and efficacy of the technology, thereby facilitating its broad application in non-invasive medical fields;

6. Cross-disciplinary integration. Currently, most research is primarily focused on a single field, such as sensors or wound dressings. In the future, research can be conducted to develop multifunctional nanofibers through cross-disciplinary integration. For example, by preparing multi-layered nanofibers, various functions can be integrated. For instance, nanofibers can be developed that possess both sensing capabilities and drug-release capabilities. These nanofibers can be used to monitor wound healing processes in real time while slowly releasing antibiotics to prevent infection. Developing nanofiber materials with temperature-regulating functions along with antibacterial and antiviral properties is also possible. For example, temperature regulation can be achieved by incorporating phase-change materials (PCMs) into nanofibers and using antibacterial agents such as silver nanoparticles or copper nanoparticles to impart antibacterial and antiviral functions to the material. Through these optimizations and expansions, the application and development of electrospun nanofiber technology in non-implant medical fields can be better promoted.

With these comprehensive summaries, this paper underscores the extensive applicability and developmental potential of electrospun nanofibers in the non-invasive medical sector, aiming to provide valuable insights for future research and clinical practice.

## Data Availability

No new data were created or analyzed in this study. Data sharing is not applicable to this article.
